# Relationship between dental and periodontal health status and the salivary microbiome: bacterial diversity, co-occurrence networks and predictive models

**DOI:** 10.1038/s41598-020-79875-x

**Published:** 2021-01-13

**Authors:** M. Relvas, A. Regueira-Iglesias, C. Balsa-Castro, F. Salazar, J. J. Pacheco, C. Cabral, C. Henriques, I. Tomás

**Affiliations:** 1 Institute of Research and Advanced Training in Health Sciences and Tecnologies (IINFACTS), IUCS-Cespu-Instituto Universitário de Ciencias da Saúde, Gandra, Paredes, Portugal; 2 Oral Sciences Research Group, Special Needs Unit, Department of Surgery and Medical-Surgical Specialties, School of Medicine and Dentistry, Health Research Institute of Santiago (IDIS), Universidade de Santiago de Compostela, Galicia, 15872 Santiago de Compostela, Spain

**Keywords:** Bacteria, Clinical microbiology, Microbial communities, Pathogens

## Abstract

The present study used 16S rRNA gene amplicon sequencing to assess the impact on salivary microbiome of different grades of dental and periodontal disease and the combination of both (hereinafter referred to as oral disease), in terms of bacterial diversity, co-occurrence network patterns and predictive models. Our scale of overall oral health was used to produce a convenience sample of 81 patients from 270 who were initially recruited. Saliva samples were collected from each participant. Sequencing was performed in Illumina MiSeq with 2 × 300 bp reads, while the raw reads were processed according to the Mothur pipeline. The statistical analysis of the 16S rDNA sequencing data at the species level was conducted using the phyloseq, DESeq2, Microbiome, SpiecEasi, igraph, MixOmics packages. The simultaneous presence of dental and periodontal pathology has a potentiating effect on the richness and diversity of the salivary microbiota. The structure of the bacterial community in oral health differs from that present in dental, periodontal or oral disease, especially in high grades. Supragingival dental parameters influence the microbiota’s abundance more than subgingival periodontal parameters, with the former making a greater contribution to the impact that oral health has on the salivary microbiome. The possible keystone OTUs are different in the oral health and disease, and even these vary between dental and periodontal disease: half of them belongs to the core microbiome and are independent of the abundance parameters. The salivary microbiome, involving a considerable number of OTUs, shows an excellent discriminatory potential for distinguishing different grades of dental, periodontal or oral disease; considering the number of predictive OTUs, the best model is that which predicts the combined dental and periodontal status.

## Introduction

Oral diseases are a major public health concern, having a negative effect on individuals, communities and society at large^1^. Recent data from the Global Burden of Disease Study (GBD) revealed that around 3.5 billion people worldwide have suffered from an untreated dental condition, mainly in the form of caries, periodontitis, tooth loss and edentulism^2^. It is also well-known that oral diseases such as periodontitis have significant associations with chronic systemic conditions like cardiovascular disease and diabetes, with poor oral health having an adverse impact on the development and control of these types of disorder^3–5^.

Several sets of diagnostic criteria have been developed over the last few decades for use in the classification of dental caries. These include: the Decayed, Missing and Filled Teeth (DMFT) index^6^; the Dundee Selectable Threshold Method (DSTM)^7^; the International Caries Detection and Assessment System (ICDAS)^8^; and the Caries Assessment Spectrum and Treatment (CAST) tool^9^. The Community Periodontal Index of Treatment Needs (CPITN)^10^ and the recent International Classification of Periodontal and Peri-implant Diseases and Conditions are also used to categorise periodontal status, with the latter distinguishing forms of the disease based on a multidimensional staging and grading system^11,12^.

In recent years, scales have been designed based on a combination of different dental and/or periodontal parameters. These include: the Total Dental Index (TDI)^13^; the Modified Total Dental Index (MTDI)^14,15^; the Asymptotic Dental Score (ADS)^16^; and the Brief Oral Health Status Examination (BOHSE)^17^. In 2013, our group developed a new scale of overall oral health, combining parameters relating to caries, gingivitis and periodontitis. The aim of this clinical scale, which is applicable to the adult population, is to obtain a single numerical value that synthesises the patient's oral health status, allowing the establishment of an individualised grade of dental or periodontal health^18^.

A symbiotic relationship between resident oral microbiota and a host is essential for the maintenance of oral health^19,20^. Indeed, several studies using 16S rRNA gene next-generation sequencing (NGS) have shown that local compositional changes in the bacterial communities, which are associated with dental caries and periodontitis, are far more complex than previously believed^21,22^. Assuming saliva may reflect the presence of oral disease^23^, investigations using 16S rRNA gene NGS have been conducted to identify which salivary microbiota profiles are related to oral health^24^, dental caries^25^ and periodontitis^26^.

However, very few 16S-based studies have analysed the impact of various dental and periodontal parameters simultaneously to demonstrate which of them have the greatest repercussions for the salivary microbiota^27–29^. As a consequence, our study used 16S rRNA gene NGS to examine the bacterial diversity and the co-occurrence network patterns of the salivary microbiota in patients who have been classified clinically by our self-designed and previously validated scale of overall oral health, including dental and periodontal parameters. Also, another objective was to evaluate the diagnostic potential of salivary microbiome to discriminate between different clinical conditions.

## Materials and methods

### Selection of the study group

The participants in our study were recruited between January, 2016 and July, 2017 from a group of patients aged between 25 and 65 who had visited the Dental Clinic of Instituto Superior de Ciências da Saúde Norte, Cooperativa de Ensino Superior, Politécnico e Universitário (CESPU) (Gandra, Paredes, Portugal) for a dental check-up.

The following exclusion criteria were applied: (1) fewer than 18 teeth; (2) the presence of removable dental prostheses or orthodontic devices; (3) antibiotic treatment or the routine use of oral antiseptics in the previous 3 months; and (4) the incidence of any etiological factors (systemic diseases, medication, radiotherapy etc.) that could trigger changes in the production and/or composition of saliva^30^. After applying these criteria, 270 patients remained in our sample. The research was conducted based on the principles of the Declaration of Helsinki (revised in 2000) concerning studies involving human experimentation^31^. The research protocol was approved by the Instituto Superior de Ciências da Saúde–Norte, CESPU (registration number 35/CE-IUCS/2019), and all the participants provided their written informed consent to their involvement in the study.

### Application of our oral health scale

Examination of the oral cavity (excluding the third molars) was performed by a single trained examiner using a conventional dental mirror, a probe, and a CP11 manual periodontal probe calibrated at 3, 6, 8 and 11 mm. Six sites per tooth were examined, taking into account the dental and periodontal variables included in our new scale. The findings are expressed in terms of the number of sites revealing specific oral conditions, and were evaluated according to tested and reproducible indices.

The following four clinical parameters were utilised: (1) the number of sites with supragingival plaque, based on the O'Leary Plaque Index^32^; (2) the number of sites with caries and their severity (1 = affecting the enamel, 2 = affecting the enamel and dentine, and 3 = affecting the enamel, dentine and pulp); (3) the number of sites with gingival inflammation, based on the Ainamo and Bay Gingival Index^33^; and (4) the number of sites with periodontal pockets ≥ 4 mm and their severity (average depth of the pockets).

The participants’ dental and periodontal health grades (DG and PG, respectively) corresponded to the grades assigned to at least two of the three variables analysed in each of these categories. If there were differences between the grades allocated to each of the variables in a category, the parameters for "number of caries" and "number of periodontal pockets ≥ 4 mm" took precedence. If the same grade was allocated to two variables in a category, but the third variable’s grade was two levels higher, the value assigned to it was one grade higher than that of the matching variables. The oral health grade (oral grade—OG) was determined by the category (dental or periodontal) with the highest ranking, enabling patients to be classified based on the score for their dental and periodontal health and the combination of both conditions (Table 1).

**Table 1:** Scale of overall oral health, involving grades of dental and periodontal health.

	Grade 0	Grade 1	Grade 2	Grade 3
**Grades of dental health**				
Supragingival plaque (O’Leary index)	0	1–56	57–112	> 112
Caries	0	1–4	5–8	≥ 9
Severity of the caries (median)	0	1	2	3
**Grades of periodontal health**				
Gingival inflammation (Ainamo and Bay index)	0	1–56	57–112	> 112
Periodontal pockets ≥ 4 mm	0	1–56	57–112	> 112
Severity of the pockets (mean)	< 4 mm	4–4.9 mm	5–5.9 mm	≥ 6 mm

Our scale of overall oral health was used to select a convenience sample of 81 patients from the 270 initially recruited. These 81 participants were given the following OGs: 0 for 17 of them; 1 for 25; 2 for 28; and 3 for 11. In relation to the subscales, 47 patients had a PG of 0 and different DGs, and 46 had a DG of 0 and different PGs.

### Collection of saliva samples and the 16S rDNA gene amplicon sequencing

Unstimulated saliva samples (2–3 mL) were collected from each participant using the spitting method^34^. None of the subjects had eaten or brushed their teeth for at least 1 h before they provided their sample.

Total DNA was extracted from the saliva using a commercial kit (QIAamp DNA Mini kit; Qiagen, GmbH, Hilden, Germany) and according to the manufacturer's instructions, albeit with minor modifications, including the addition of a lysozyme treatment (20 mg/ml at 37 ºC for 30 min). The isolated DNA was eluted in 200 µl of distilled and apyrogenic water, and its quality and concentration were assessed using a Nanodrop spectrophotometer (ND-2000 Spectrophotometer, Wilmington, USA). DNA samples with spectrophotometer ratios (Abs 260/280) between 1.5 and 2.0 were considered to be acceptable for inclusion in the study^35^. We used the ZymoBIOMICS Microbial Community DNA Standard (Catalog No. D6306, Zymo Research, Irvine, CA, USA), which is a mix of genomic DNA isolated from pure cultures of eight bacterial and two fungal strains, as both the bacterial mock community and the positive control for the downstream procedures. Mock DNAs were amplified and sequenced in the same way as all the other samples used in the experiment.

A polymerase chain reaction (PCR) amplification of the 16S rDNA gene was performed with the KAPA HiFi HotStart DNA Polymerase KAPA HiFi HotStart ReadyMixPCR Kit (Cat. No. KK2602, 7958935001; Kapa Biosystems, F. Hoffmann-La Roche Ltd, Basel, Switzerland). The V3–V4 hypervariable region was amplified as previously described^36^ using the following primers in a limited-cycle PCR:

- V3-V4-Forward (5′-CCTACGGGNGGCWGCAG-3).
- V3-V4-Reverse (5′-GACTACHVGGGTATCTAATCC-3).

A set of modified primers, V3-V4-F and V3-V4-R, were also used. This set contained a 1–3 bp "heterogeneity spacer" that we designed to mitigate the issues caused by low sequence diversity amplicons.

Each PCR amplification was carried out on a total volume of 10 µl, which comprised 4 µl of DNA, 0.2-μM from each forward and reverse primer, and a Kapa ready mix (Kapa Biosystems). The PCR conditions were modified by conducting: (1) an initial denaturation at 95 °C for 3 min; (2) 25 three-step cycles of 95 °C for 30 s, 55 °C for 30 s and 72 °C for 30 s; and (3) a final 5 min extension at 72ºC. Water, up to a total volume of 50 μl, was added after the first PCR step. The reactions were purified using AMPure XP beads (Beckman Coulter, Brea, CA, USA) with a 0.9X (V3-V4 amplicon) ratio, according to the manufacturer's instructions. PCR products were eluted from the magnetic beads with 32 μl of Buffer EB (Qiagen N.V, Hilden; Germany), with 30 μl of the eluate transferred to a fresh 96-well plate. The primers described above contain overhangs that enable the addition of full-length Nextera adapters. Barcodes are available for multiplex sequencing in a second PCR step, which produces sequencing-ready libraries. To this end, 5 μl of the first amplification was used as a template for the second PCR, with Nextera XT v2 adaptor primers added up to a final volume of 50 μl. The PCR mix and thermal profile employed for the first PCR were also used for the second, but only for eight cycles. After the second PCR, 25 μl of the final product was purified and normalised with the SequalPrep normalisation kit (Invitrogen, Carlsbad, CA, USA), according to the manufacturer's protocol. Libraries were eluted in a 20-μl volume and pooled for sequencing.

Final pools were quantified with a qPCR using the Kapa library quantification kit for Illumina Platforms (Kapa Biosystems) on an ABI 7900HT real-time cycler (Applied Biosystems, Foster City, CA, USA). Sequencing using v3 chemistry with a loading concentration of 18 pM was performed in Illumina MiSeq (Illumina Inc., San Diego, CA, USA) with 2 × 300 bp reads. In all cases, 10% of the PhIX control libraries were spiked to increase the diversity of the sequenced samples.

In parallel, negative control tests of the sample-collection buffer, DNA-extraction and PCR-amplification steps were conducted routinely under the same conditions and using reagents. One such non-template control was subjected to the library preparation and then sequenced. As expected, this yielded very few reads (220 per sample), in contrast to an average of 188,713 reads/library in the sample-derived collections. The sequences obtained were deposited in the SRA database under accession number PRJNA623352.

### Bioinformatic processing

The raw reads were obtained in the fastq format and processed according to the Mothur pipeline proposed by Schloss et al.^37^, albeit with some modifications. Four samples with a very low number of raw sequences were removed (fewer than 436 per sample). The final raw-read total was 15,285,797 (mean ± standard deviation per sample = 188,713 ± 37,473, median = 189,918, maximum–minimum number of sequences in a sample = 282,936–119,882).

The Needleman-Wunsch alignment algorithm was used to obtain the contigs from the raw sequences^38^. The quality filtering criteria applied were contigs with: (1) errors in the primers; (2) ambiguous bases; (3) more than eight homopolymers; (4) mismatches; and (5) overlap of less than 25 bp. Lengths below 400 bp or greater than 535 bp were discarded.

The unique candidate sequences were aligned against the SILVA-based bacterial reference alignment^39^, again using the Needleman-Wunsch algorithm^38^. A total of 52,544 potential chimeras were detected with the VSEARCH tool^40^, representing 4.06% of the total unique sequences. The final total of high-quality reads was 4,269,754.

The opticlust algorithm and the Matthews Correlation Coefficient Metric (MCC) were used to group the sequences into operational taxonomic units (OTUs) with a similarity of 97%^41^. A total of 3788 OTUs were obtained from all the samples, and the native Bayesian classifier proposed by Wang et al.^42^ was employed to categorise them. The Human Oral Microbiome Database, version 15.2, was used for this purpose^43^ and includes 1015 different consensus strains. A classification threshold of 80% was employed for the taxonomic designations at all levels. The non-classified level was assigned the term "unclassified".

The mock D6306 sample was processed using the same protocol to evaluate the quality of the sequencing and the bioinformatic pipeline. First, a reference database was constructed from the 58 different 16S rRNA sequences provided by the manufacturer. These corresponded to all the variants of the 16S rRNA genes of the eight species included in the sample. The mock sample was then extracted from the pipeline and analysed separately against the reference database, producing errors of 0.0000073% in the bp from a total of 57,350 sequences with a length of 400 bp. Fourteen OTUs were obtained from the mock sample, with eight of them highly abundant (between 4241 and 14,971 counts) and present in very similar numbers to the theoretical percentages indicated by the manufacturer. Only six OTUs were present in very low abundances (one or two counts), which could be due to sequencing errors. As a consequence, the protocol applied was considered to be of high quality.

### Statistical analysis

The statistical analysis of the 16S rDNA sequencing data at the species level was performed according to the protocol recently proposed by McMurdie and Holmes^44^, using implementations in R such as the phyloseq, DESeq2 and microbiome packages (versions 1.34.0, 1.30.0 and 1.12.0, respectively)^45–47^. An independent filter had previously excluded from the statistical analysis the OTUs with an abundance of ≤ 2 counts and a presence in ≤ 3 samples (3.89%)^48^, leaving a final total of 403 OTUs(the final number of high-quality reads was 4,235,218).

The impact of the different oral-health scale grades (DG, PG and OG) on the salivary microbiome was investigated in relation to: (1) four indicators of alpha diversity and the structure of the bacterial community; and (2) the composition of the core microbiota and the testing of differential abundances. The individualised impact of the dental subscale was analysed in the participants with a PG of 0 while the periodontal subscale was assessed in those with a DG of 0. Four different comparative analyses of the grades for the dental and periodontal subscales and scale of overall oral health were performed: grade 0 *vs* 123; grade 0 *vs* 1; grade 0 *vs* 23; and grade 1 *vs* 23.

#### Alpha diversity indicators and structure of the salivary bacteria community

The phyloseq and microbiome packages were used in the following ways to obtain data relating to the four alpha diversity indicators^45,47^: (1) taxa richness: the absolute count data ("observed") and the coverage index; the latter specifies how many of the more abundant OTUs are required to achieve a particular proportion of the occupied ecosystem (95%); and (2) diversity and evenness of the taxa present in the samples: the Shannon and Pielou indices^49,50^. The Student t-test was used to conduct different comparative analyses.

A principal coordinate analysis (PCoA) was employed to visualise the clustering of the salivary samples in relation to their respective dental, periodontal or oral health grades. The "ape" package, version 5.3^51^, was used to rotate the phylogenetic tree, while the weighted unifrac algorithm^52^ was applied in the phyloseq package to obtain the phylogenetic distance matrix. A non-parametric permutational multivariate analysis of variance (PERMANOVA)^53^ was used to measure the multivariate community-level differences between the groups. These analyses were performed using the vegan package, version 2.5.6^54^.

#### Composition of the core salivary microbiome and testing the differential abundances

We used the microbiome package^47^ to identify the central taxa present with a prevalence rate of 100% for each grade, or combination of grades from the dental and periodontal subscales and the oral scale.

The DESeq2 package^46^ was used to identify the bacteria with the most significant changes in differential abundance at the species level for the different conditions. Improvements to the stability and dispersion of the counts (variance) were required before it was possible to calculate the differential abundances for the different species present in the samples being compared. To this end, we used the estimate SizeFactors function in DESeq2^46^ to transform the stabilisation of the variance. The differential abundances were measured with the log2foldchange value, and the different conditions were compared using the Wald test with the Benjamini–Hochberg correction (Q parameter = 0.1, FDR < 10%). The differential-abundance measurements were statistically significant if the adjusted p-value was < 0.05 (− log10 adjusted p-value = 1.3).

#### Co-occurrence networks in the salivary microbiome

After filtering out the OTUs with > 10 counts in the total samples of each clinical group, we conducted an analysis of the co-occurrence networks for OG0, OG23, DG23 and PG23. The SparCC method was used to generate the networks^55^, as this has been shown to enable researchers to detect the linear relationships in a compositional dataset to a high degree of precision^56^.

Default parameters and the SpiecEasi package (version 1.1.0) were employed to run SparCC^57^, and the correlation matrix we obtained was filtered using an absolute correlation score greater than or equal to 0.5. The networks were subsequently visualized using the igraph package (version 1.2.4.1)^58^, where each node represents one OTU and each edge the correlations between the OTU abundances.

A set of measures was calculated to describe the topology of the resulting networks: the number of nodes and edges; the density; the average number of neighbours; the characteristic path length; the clustering coefficient, also known as transitivity; the centralization; the modularity; the number of subnetworks; and the number of modules^59^.

We calculated the Betweenness Centrality (BC)^60^ to measure the relative importance of each taxon within the network (how influential a taxon is within a network). This determines the fraction of the shortest paths through one particular bacterial taxon to another. The BC of a taxon in a network reflects the importance of the control the taxon exerts over the interactions of other taxons in the same network^60^. In line with Banerjee et al.^61^, a combined score based on a high degree value and a high BC value was used as a threshold for defining the hub or keystone OTUs in the microbial communities.

#### Diagnostic value of salivary microbiome for discriminating the clinical condition

We conducted a supervised classification in the form of a sparse partial least-squares–discriminant analysis (sPLS-DA) to facilitate the categorization of the different dental, periodontal or oral grades and identify the OTUs that best distinguished the three groups within each subscale and scale (grade 0 *vs* others, grade 1 *vs* others, grade 2, 3 *vs* others). The sPLS-DA was performed using the MixOmics package (version 1.6.3), which is dedicated to the integrative examination of “omics” data^62^. The balanced error rate (BER) was calculated for different types of distance (centroid, maximum and mahalanobis) to enable us to identify the recommended number of components for use in relation to the latent information in the OTU table.

Receiver operating characteristic (ROC) curves were constructed with the true positivity rate (sensitivity) as a function of the false positivity rate ^1-specificity^, and area under the curve (AUC) values were used to distinguish between each clinical grade. The component with the highest discrimination value was chosen in the dental and periodontal subscales and the oral health scale. It should be noted that simulations with an AUC value equal to or higher than 0.70 are generally considered to be acceptable predictive models^63^.

## Results

The 81 participants in the convenience sample had the following OG scores: 0 for 17 of them; 1 for 25; 2 for 28; and 3 for 11. Focusing on the group with the highest grade of oral pathology (OG23), we can differentiate two main subgroups: patients with DG0_PG23 (n = 14) and patients with PG0_DG23 (n = 20).

In relation to the subscales, the results were as follows: (1) 47 patients had a PG of 0 and DGs between 0 and 3 (17 had a DG of 0, nine a DG of 1, 11 a DG of 2, and 10 a DG of 3); and (2) 46 had a DG of 0 and PGs between 0 and 3 (17 had a PG of 0, 14 a PG of 1; 14 a PG of 2; and one a PG of 3). The four patients excluded due to the low number of raw sequences obtained were: two of OG1 and two of OG2 (two of DG0, one of DG1 and one of DG2; two of PG0, one of PG1 and one of PG2).

### Impact of the dental and periodontal subscales and the scale of overall oral health on the salivary microbiome: alpha diversity indicators and the structure of the bacterial community

As seen in Table 2, worsening dental or periodontal health revealed a trend of increasing alpha diversity in both the dental and periodontal subscales. In our comparisons, however, significant differences in the number of OTUs were only observed in the former: DG0 *vs* DG123 (209.82 ± 37.01 *vs* 240.57 ± 35.02; p = 0.009); and DG0 *vs* DG23 (209.82 ± 37.01 *vs* 243.55 ± 33.09, p = 0.006). Meanwhile, in our oral health scale comparisons, grade increments were linked to progressive increases in bacterial richness, especially in OG0 *vs* OG23 (no. observed OTUs = 212.52 ± 38.44 *vs* 240.24 ± 32.77, p = 0.015; coverage index 95% = 65.17 ± 15.59 *vs* 78.24 ± 20.42, p = 0.013). This was also the case for the Shannon Index values (OG0 = 3.67 ± 0.23, and OG23 = 3.83 ± 0.26, p = 0.026).

**Table 2:** Alpha diversity indicators in the different grades of the dental and periodontal subscales, as well as in the scale of overall oral health.

	No. observed OTUs	Coverage index (95%)	Shannon index	Pielou index
**Dental health grade (DG)**				
DG0	209.82 ± 37.01	65.11 ± 15.64	3.66 ± 0.23	0.68 ± 0.03
DG123	240.57 ± 35.02	74.64 ± 20.54	3.79 ± 0.24	0.69 ± 0.03
DG1	233.12 ± 40.84	71.37 ± 20.97	3.79 ± 0.26	0.69 ± 0.04
DG23	243.55 ± 33.09	75.95 ± 20.76	3.79 ± 0.23	0.69 ± 0.03
Significant grade comparisons (p-value < 0.05)	0 vs. 123	–	–	–
	–	–	–	–
	0 vs. 23	–	–	–
	–	–	–	–
**Periodontal health grade (PG)**				
PG0	208.52 ± 35.75	64.88 ± 15.36	3.66 ± 0.23	0.68 ± 0.03
PG123	219.48 ± 32.32	71.51 ± 18.80	3.76 ± 0.27	0.70 ± 0.04
PG1	214.61 ± 39.62	66.07 ± 18.30	3.69 ± 0.24	0.68 ± 0.03
PG23	224.00 ± 24.38	76.57 ± 18.45	3.83 ± 0.28	0.70 ± 0.04
Significant grade comparisons (p-value < 0.05)	–	–	–	–
	–	–	–	–
	–	–	–	–
	–	–	–	–
**Oral health grade (OG)**				
OG0	212.52 ± 38.44	65.17 ± 15.59	3.67 ± 0.23	0.68 ± 0.03
OG123	234.85 ± 36.47	75.28 ± 20.39	3. 80 ± 0.26	0.69 ± 0.04
OG1	226.17 ± 41.02	70.52 ± 19.86	3.76 ± 0.26	0.69 ± 0.03
OG23	240.24 ± 32.77	78.24 ± 20.42	3.83 ± 0.26	0.70 ± 0.04
Significant grade comparisons (p-value < 0.05)	0 vs. 123	0 vs. 123	0 vs. 123	–
	–	–	–	–
	0 vs. 23	0 vs. 23	0 vs. 23	–
	–	–	–	–

The following four comparisons were performed in both the dental and periodontal subscales as well as the scale of overall oral health: grade 0 vs. grade 123; grade 0 vs. grade 1; grade 0 vs. grade 23; grade 1 vs. grade 23.

**Figure 1:**
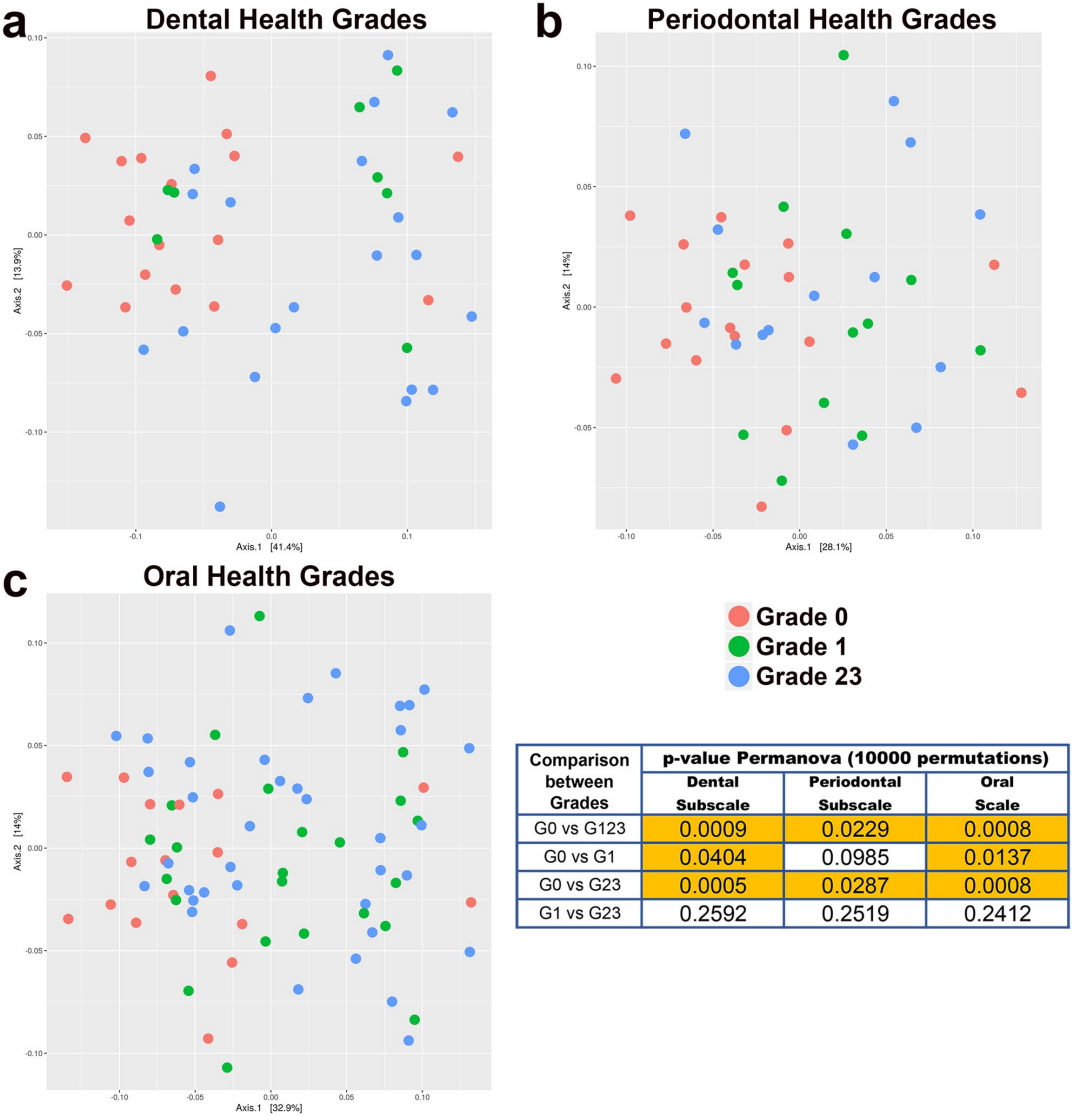
Principal Coordinate Analysis (PCoA), including PERMANOVA test values in the comparison between different grades of dental, periodontal and oral health (graphic made using the phyloseq package, version 1.34.0).

The PCoA revealed a grouping of the salivary samples taken from the participants with DGs, PGs and OGs of 0. This was in contrast to the picture for the other grades, whose compositional distributions were more diverse (Fig. 1). This visual observation was confirmed by the PERMANOVA test, which produced significant results for the comparison of grades 0 and 123 (dental subscale, p = 0.0009; periodontal subscale, p = 0.0229; oral scale, p = 0.0008). These findings were mainly at the expense of the contrast between grades 0 and 23 (dental subscale, p = 0.0005; periodontal subscale, p = 0.0287; oral scale, p = 0.0008). Focusing on the group with the highest grade of oral pathology (OG23), Permanova's test revealed that the structure of the salivary microbiome was different depending on the predominance of dental pathology (PG0_DG23) or periodontal pathology (DG0_PG23) (p = 0.027).

### Impact of the dental and periodontal subscales and the scale of overall oral health on the salivary microbiome: composition of the core microbiome and testing differential abundance

#### Composition of the core microbiome

**Figure 2:**
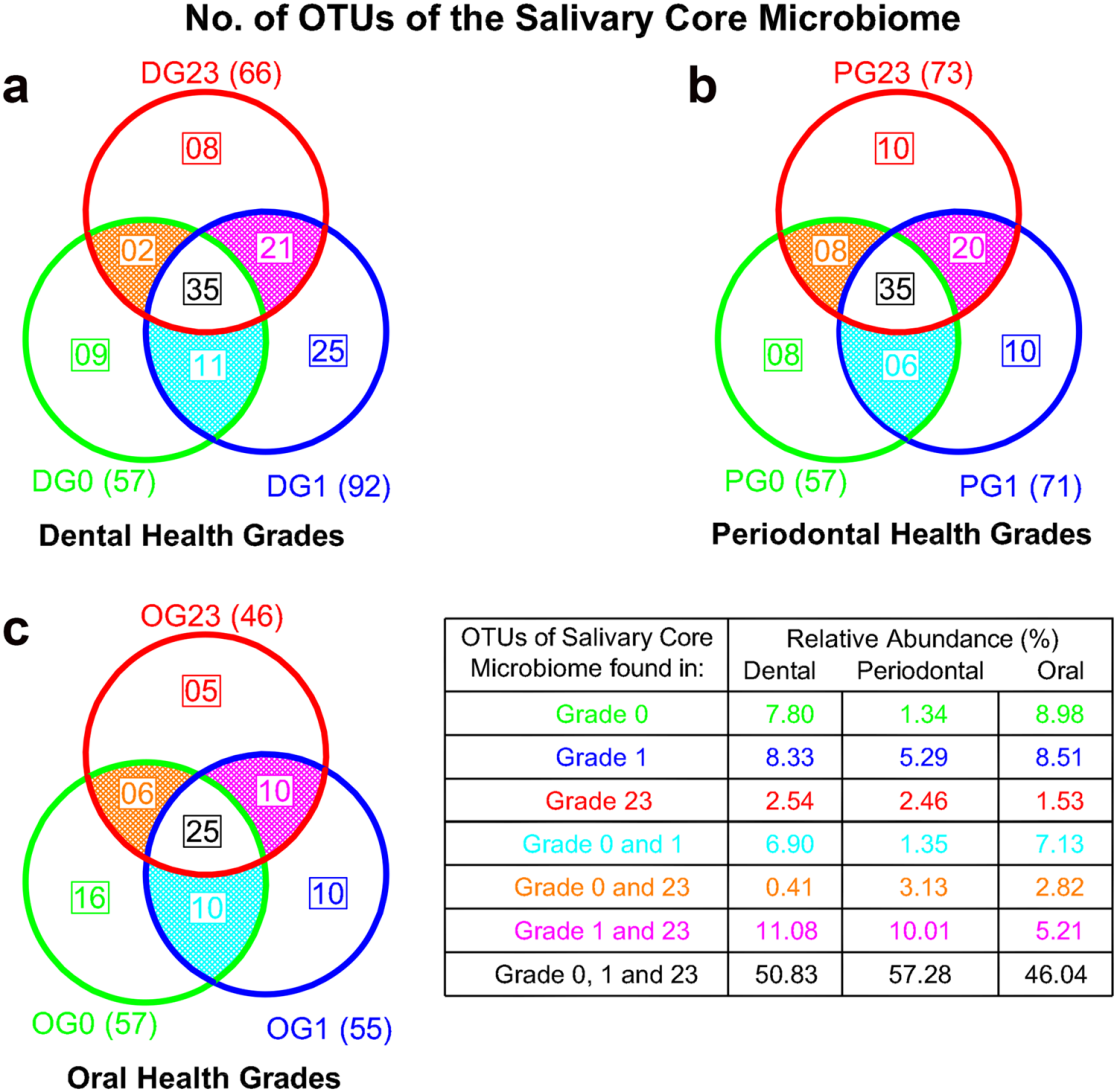
Number of OTUs of the salivary core microbiome present in different grades of dental, periodontal and oral health, as well as their respective relative abundance values.

Figure 2 uses Venn diagrams to portray the core microbiome’s number of OTUs for the different DGs, PGs and OGs. The core microbiome associated with the participants’ dental and periodontal health contained 57 species, representing 14.14% of the total number of OTUs and 63.06% of the total abundance. There were only nine taxa in DG0 and eight in PG0 (the specific core of grade 0), exemplifying abundances of 7.80% and 1.34%, respectively. Of these specific core species, five were common to both the dental and periodontal health conditions: *Neisseria macacae, Butyrivibrio* sp. HMT_455*, Campylobacter concisus, Porphyromonas catoniae* and *Corynebacterium durum*.

There were 66 species in the core microbiome associated with the most severe dental disease (DG23) and 73 with the most serious periodontal disease (PG23), representing 16.37% and 18.11% of the total salivary microbiota, and 67.14% and 67.98% of the total abundance, respectively. There were only eight and 10 taxa present in DG23 and PG23 (the specific core of grade 23), exemplifying 2.54% and 2.46% of the abundance, respectively. Of these specific core species, only *Porphyromonas endodontalis* was common to both pathological conditions.

There were 35 taxa common to both the dental and periodontal subscales, regardless of the grade (non-specific core), representing abundances of 50.83% and 52.75%, respectively. Of these non-specific core species, 25 were common to both subscales, with the most abundant being (abundance > 1%): *Granulicatella adiacens, Haemophilus parainfluenzae, Leptotrichia* sp*., Porphyromonas pasteri, Prevotella* sp*., Prevotella melaninogenica, Prevotella salivae, Rothia mucilaginosa, Streptococcus* sp*., Streptococcus. oralis_*subsp*._dentisani_*clade_058*, Streptococcus salivarius, Veillonella* sp. and *Veillonella parvula.* Species belonging to the salivary core microbiome are shown in the supplementary data 1.

#### Testing differential abundance

The results for the dental health subscale revealed differential abundances for the different grades in 102 species (25.31% of the total OTUs), 39 of which were core species and 63 non-core (9.67% and 15.63% of the total OTUs, respectively). There were 36 species associated with oral health and 66 with some grade of dental pathology. In the comparison of DG0 *vs* DG23, there were differential abundances in the main dental-health related OTUs (with > 1% levels in DG0; log2foldchange values ranged from 5.86 to 1.41): *Porphyromonas pasteri, Fusobacterium periodonticum, Veillonella parvula, Alloprevotella* sp. HMT 473*, Alloprevotella tannerae* and *Neisseria subflava*. Those related to high grades of dental pathology (> 0.5% levels in DG23; log2foldchange values ranged from − 1.98 to − 1.35) were: *Alloprevotella* sp*.* HMT 308*, Streptococcus parasanguinis* clade 411*, Atopobium* sp*., Fusobacterium nucleatum_*subsp*._vincentii, Megasphaera micronuciformis* and *Alloprevotella rava* (Table 3).

**Table 3:**
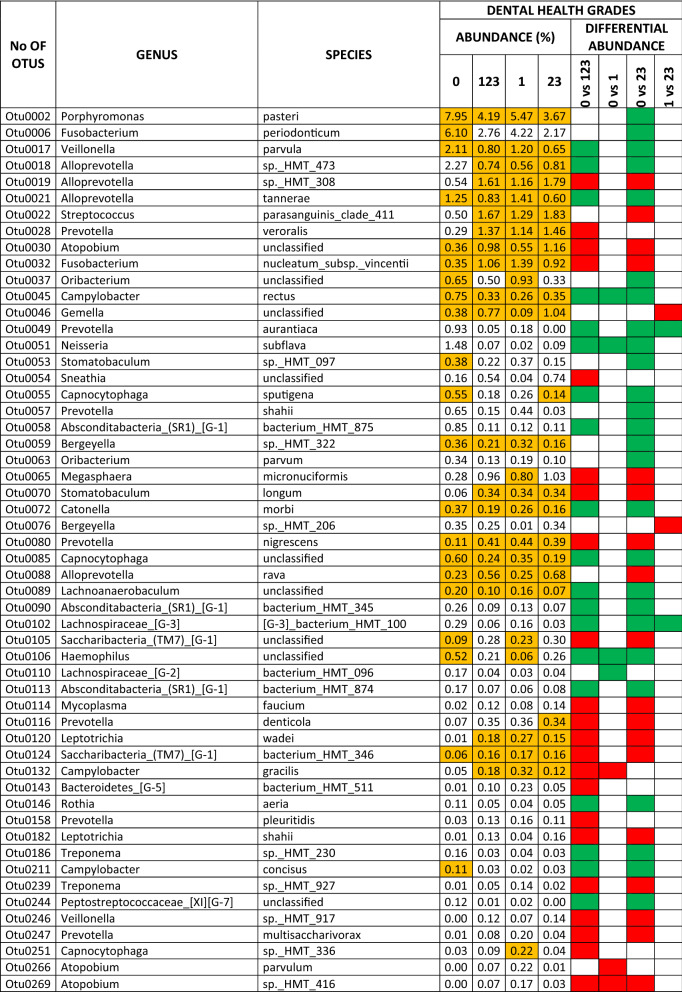
Species that presented differential abundance among different grades of dental health, and exhibited an abundance > 0.1% (green square, in favour of health; red square, in favour of pathology). The species that belonged to the core are colored orange.

The results for the periodontal health subscale revealed differential abundances for the different grades in 27 species (6.69% of the total OTUs), eight of which were core species and 19 non-core (1.98% and 4.71% of the total OTUs, respectively). Twelve species were associated with oral health and 15 with some grade of periodontal pathology. In the comparison of PG0 *vs* PG23, there were differential abundances in the two main periodontal-health related OTUs (with > 1% levels in PG0): *Haemophilus parainfluenzae* and *Capnocytophaga leadbetteri*, with log2foldchange values of 1.16 and 2.31, respectively. Those associated with high grades of periodontal pathology (with > 0.1% levels in PG23; log2foldchange values ranged from − 3.07 to − 2.13) were: *Tannerella forsythia*, *Mycoplasma faucium*, *Fretibacterium* sp., and *Bacteroidetes* [G-5] bacterium HMT 511 (Table 4).

**Table 4:**
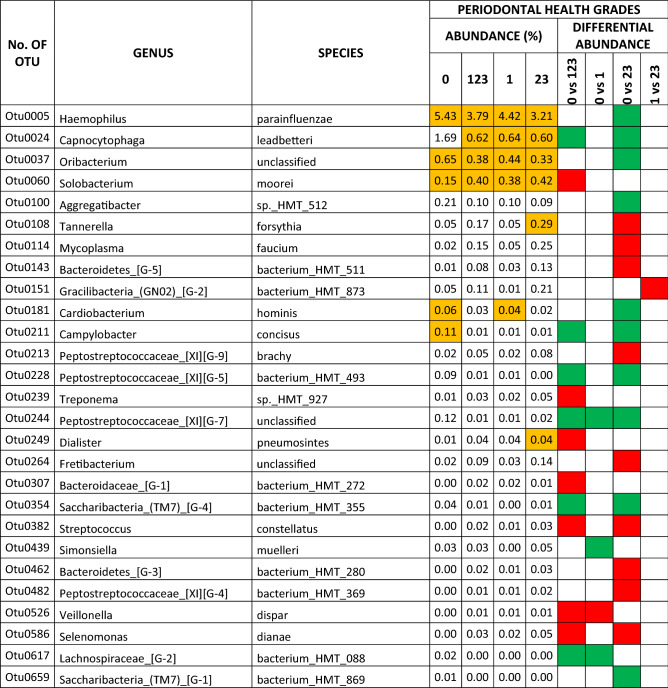
Species that presented differential abundance among different grades of periodontal health (green square, in favour of health; red square, in favour of pathology). The species that belonged to the core are colored orange.

The results for the scale of overall oral health revealed differential abundances for the different grades in 88 species (21.83% of the total OTUs), 22 of which were core species and 66 non-core (5.45% and 16.37% of the total OTUs, respectively). There were 22 species associated with oral health and 66 with some grade of oral pathology, including dental and periodontal conditions. In the comparison of OG0 *vs* OG23, the differential abundances in the main oral-health related OTUs (with > 1% levels in OG0; log2foldchange values ranged from 1.71 to 0.88) were: *Porphyromonas pasteri, Haemophilus parainfluenzae, Veillonella parvula,* and *Capnocytophaga leadbetteri*. Those associated with high grades of oral pathology (> 1% levels in OG23; log2foldchange values ranged from − 2.86 to − 1.33) were: *Alloprevotella* sp. HMT 308*, Fusobacterium nucleatum_*subsp*._vincentii* and *Porphyromonas gingivalis* (Table 5). The relative abundances of total OTUs in dental, periodontal, oral health grades, and analyses of differential abundance between grades are detailed in the supplementary datas 2–4.

**Table 5:**
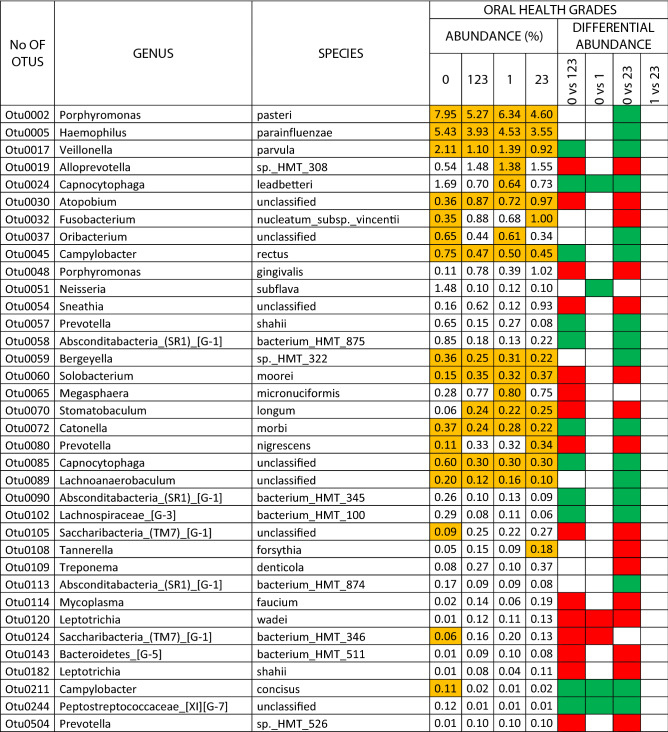
Species that presented differential abundance among different grades of the scale of overall oral health, and exhibited an abundance > 0.1% (green square, in favour of health; red square, in favour of pathology). The species that belonged to the core are colored orange.

### Impact of the dental and periodontal subscales and the scale of overall oral health on the salivary microbiome: co-occurrence network patterns and discriminatory potential of the salivary microbiome

#### Co-occurrence network patterns

Table 6 shows the topological parameters of the co-occurrence networks in the different clinical groups. In general terms, the most important differences were detected between OG0 and OG23 followed by OG0 and DG23.

**Table 6:** Topological parameters of the co-occurrence networks in the different grades of the dental and periodontal subscales, as well as oral health scale.

	Oral health grade		Dental health grade	Periodontal health grade
	OG0	OG23	DG23	PG23
Network coverage^a^	65.75%	43.42%	70.71%	77.17%
Number of nodes	265	175	285	311
Number of edges	1867	436	1447	2697
Number of positive correlations (%)	1055 (56.5%)	362 (83.1%)	915 (63.2%)	1468 (54.4%)
Number of negative correlations (%)	812 (43.5%)	74 (16.9%)	532 (36.8%)	1229 (45.6%)
Ratio of positive correlations and nodes	3.98	2.06	3.21	4.72
Ratio of negative correlations and nodes	3.06	0.42	1.86	3.95
Density	0.053	0.028	0.0357	0.055
Average number of neighbors	15.09	5.98	11.15	18.34
Characteristic path length	2.86	4.27	3.10	2.67
Clustering coefficient	0.37	0.37	0.30	0.35
Centralization	0.16	0.08	0.09	0.14
Modularity	0.36	0.62	0.36	0.33
Number of subnetworks	2	5	3	1
Number of modules	28	19	26	29
Number of modules with more than 3 nodes	9	9	4	9

^a^Percentage of OTUs present in the co-occurrence network with respect to the total number of OTUs.

The network coverage in OG0 was higher than in OG23 (65.75% and 43.42%, respectively), as was the number of edges (1867 and 436, respectively). There was also a better balance between the number of positive and negative correlations in OG0 with respect to OG23 (56.5% and 43.5% and 83.1% and 16.9%, respectively). The network in OG0 had a higher density, average number of neighbours, and higher centralization values than the network in OG23 (0.05, 15.09 and 0.16 *vs* 0.02, 5.98 and 0.08); in contrast, the characteristic path lengths and modularity scores were lower (2.86 and 0.36 in OG0 *vs* 4.27 and 0.62 in OG23). The OG0 network had fewer subnetworks and a higher number of modules (two and 28 *vs* five and 19 in OG23). All of these differences (except for the modularity values) were also observed between the OG0 and DG23 networks, albeit to a lesser extent. In contrast, all of the other parameters had similar values in the comparison between OG0 and PG23.

**Figure 3:**
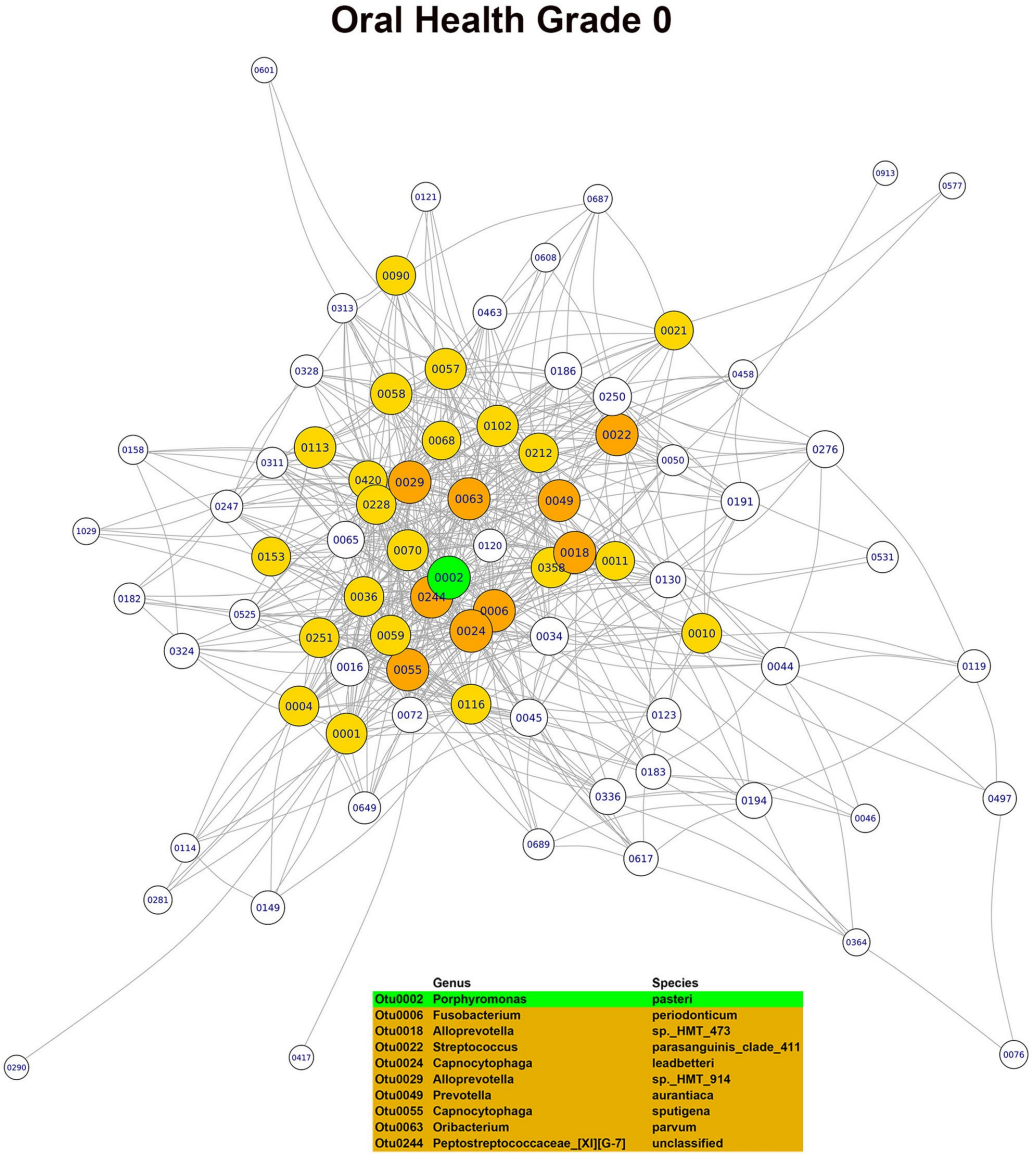
Main module of the co-occurrence network associated with grade 0 of the overall oral health scale (node = 79; degree = 1723) (graphic made using the igraph package, version 1.2.4.1).

**Figure 4:**
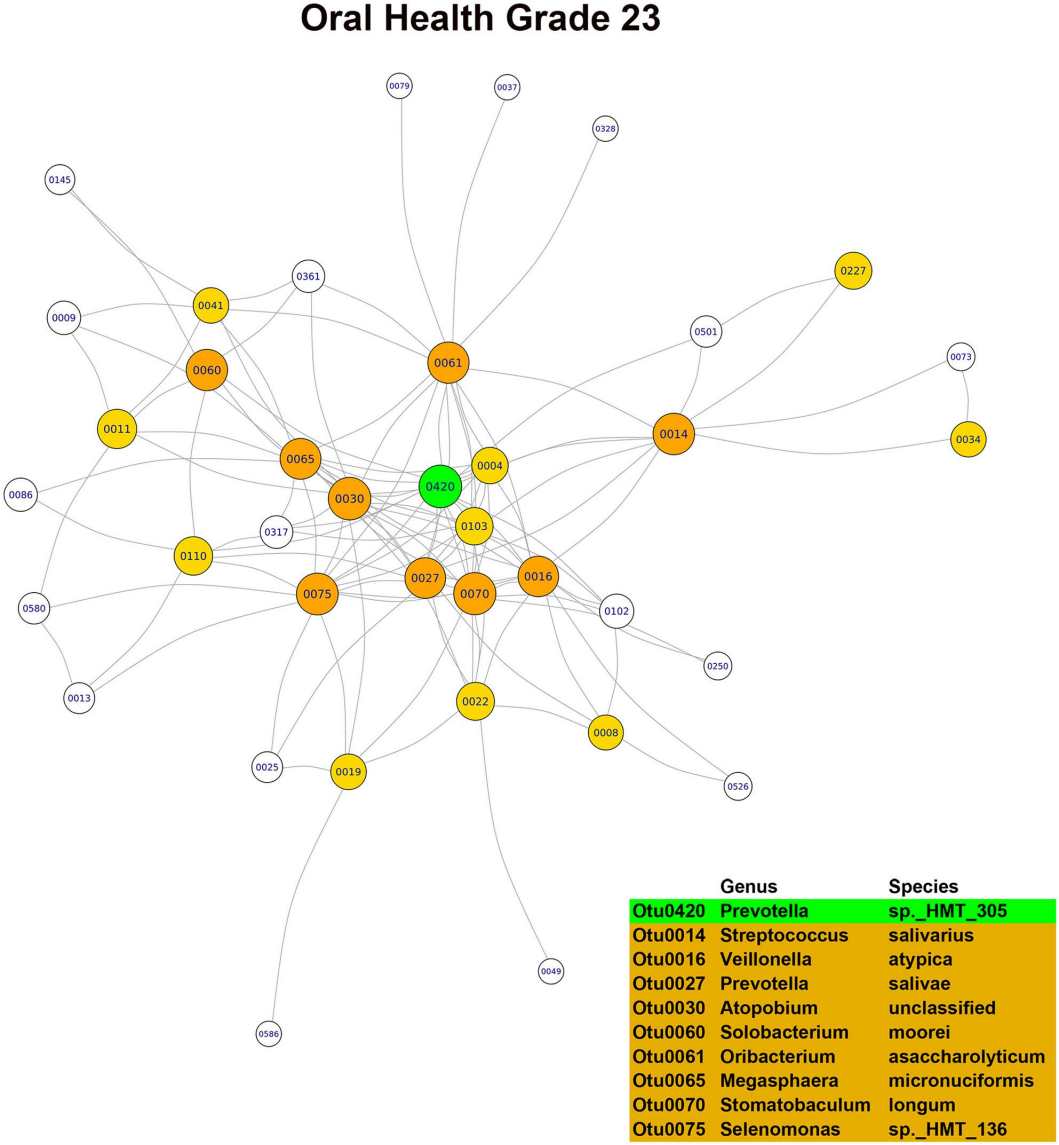
Main module of the co-occurrence network associated with grade 2,3 of the overall oral health scale (node = 38; degree = 280) (graphic made using the igraph package, version 1.2.4.1).

**Figure 5:**
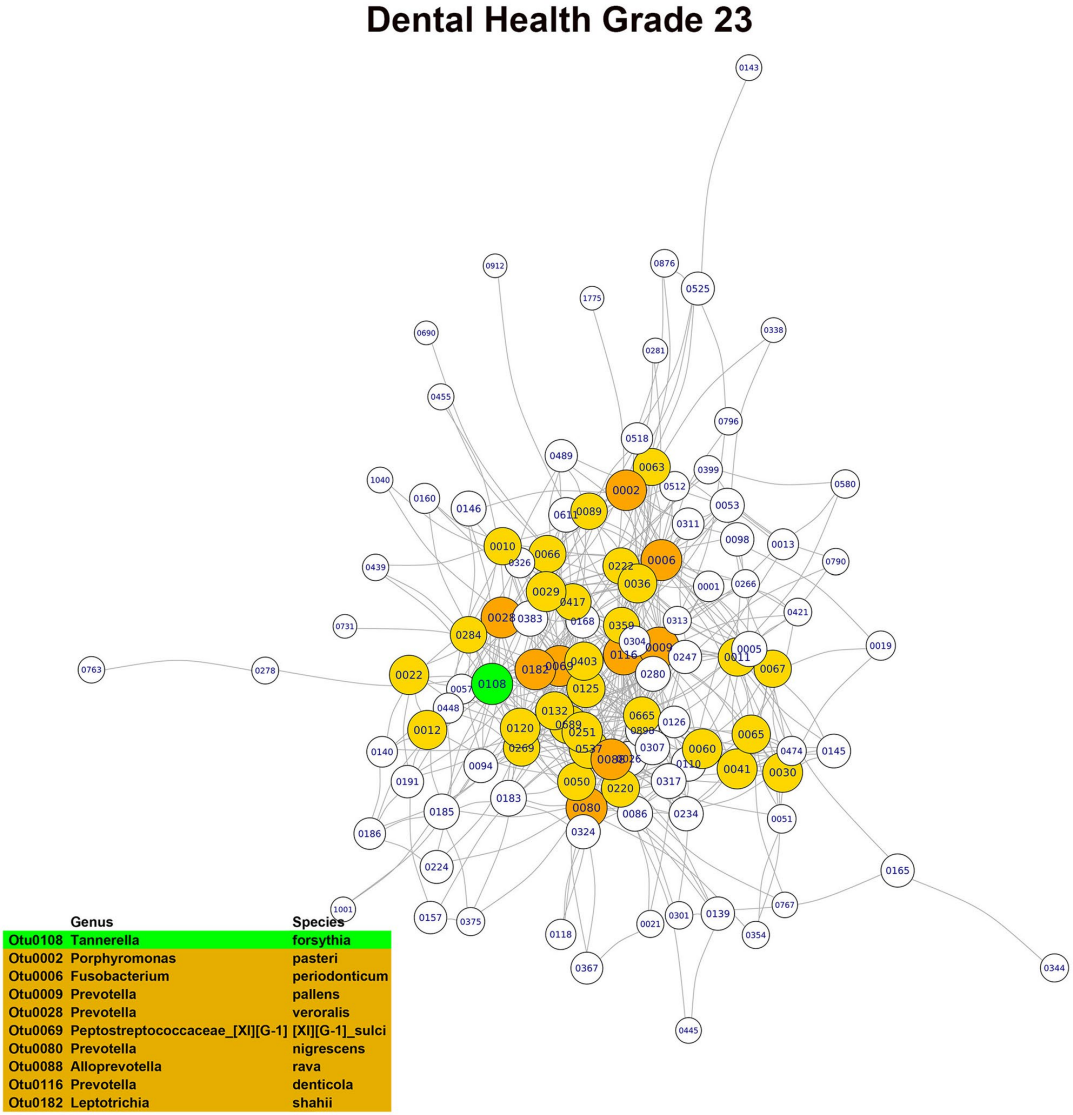
Main module of the co-occurrence network associated with grade 0 of the dental health subscale (node = 113; degree = 1444) (graphic made using the igraph package, version 1.2.4.1).

**Figure 6:**
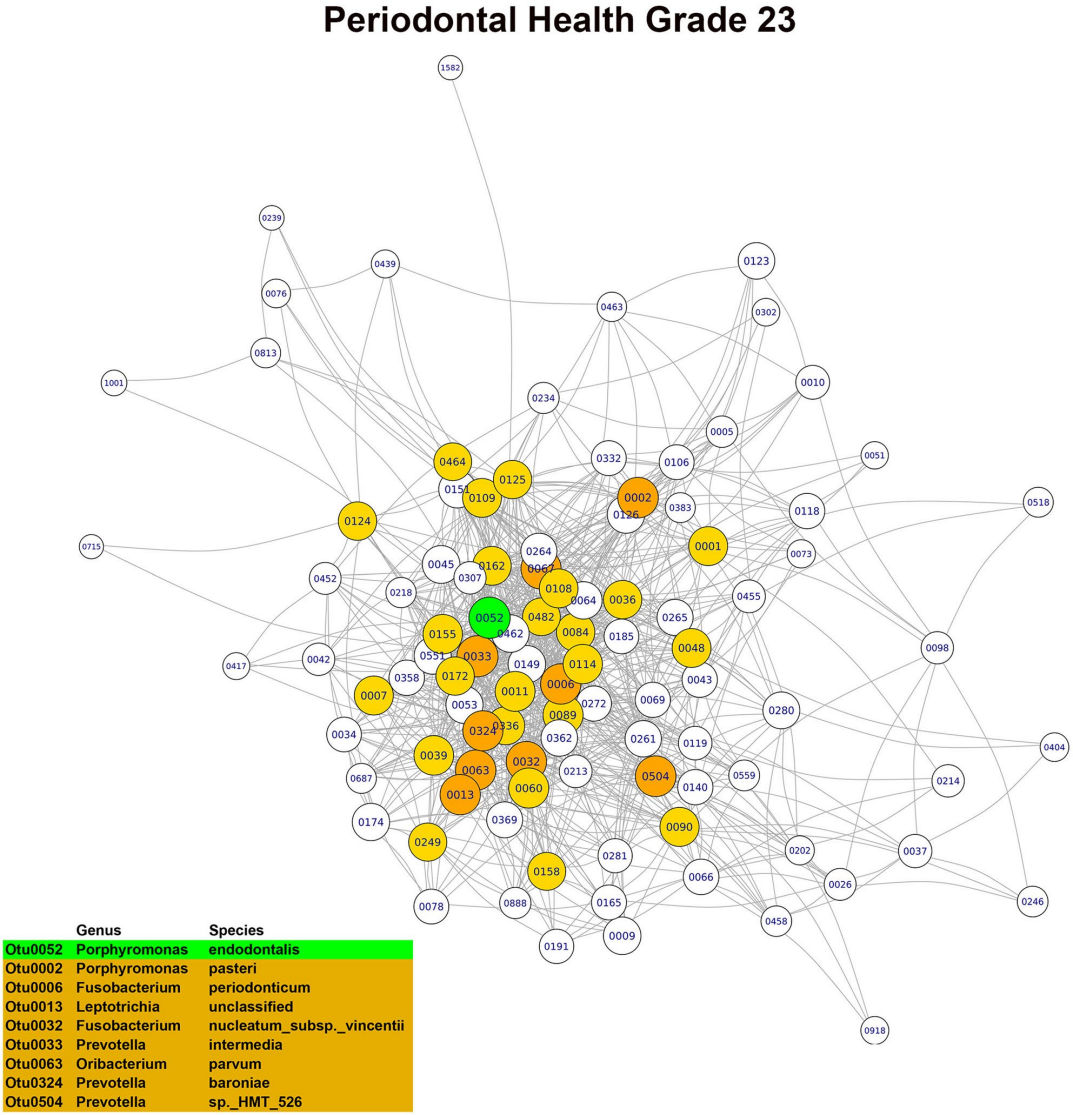
Main module of the co-occurrence network associated with grade 0 of the dental health subscale (node = 101; degree = 2663) (graphic made using the igraph package, version 1.2.4.1).

In the OG0 network, the three main hubs or keystone OTUs, based on their combined scores in the three main modules, were *Porphyromonas pasteri*, *Porphyromonas endodontalis* and *Prevotella salivae*. Although all three species were part of the core microbiome, only *Porphyromonas pasteri* was present in copious numbers (relative abundance of 8.07%) and differentially abundant in relation to the oral health status. In the OG23 network, the main hubs or keystone taxa found were *Fusobacterium periodonticum*, *Treponema socranskii* and *Prevotella* sp. *HTM 305*. Only *Fusobacterium periodonticum* was abundant (relative abundance of 2.95%) in the core microbiome, but none of the three species were differentially abundant. In the DG23 network, the main hubs or keystone taxa detected were *Tannerella forsythia*, *Fusobacterium nucleatum*_subsp. *vincentii* and *Prevotella oris*. Only *Fusobacterium nucleatum*_subsp. *vincentii* was part of the core microbiome and differentially abundant in relation to the dental disease status. In the PG23 network, the main hubs or keystone taxa detected were *Granulicatella adiacens*, *Porphyromonas endodontalis* and *Campylobacter gracilis*. Of these three species, *Granulicatella adiacens* and *Porphyromonas endodontalis* were abundant (relative abundances of 1.92% and 1.45%, respectively) and belonged to the core microbiome, but none of the three taxa were differentially abundant (Figs. 3, 4, 5, 6). Although there was a predominance of positive correlations in the keystone OTUs of the OG23 network, there were similar numbers of positive and negative associations in the keystone OTUs of the DG23 network.

#### Discriminatory potential of the salivary microbiome

**Figure 7:**
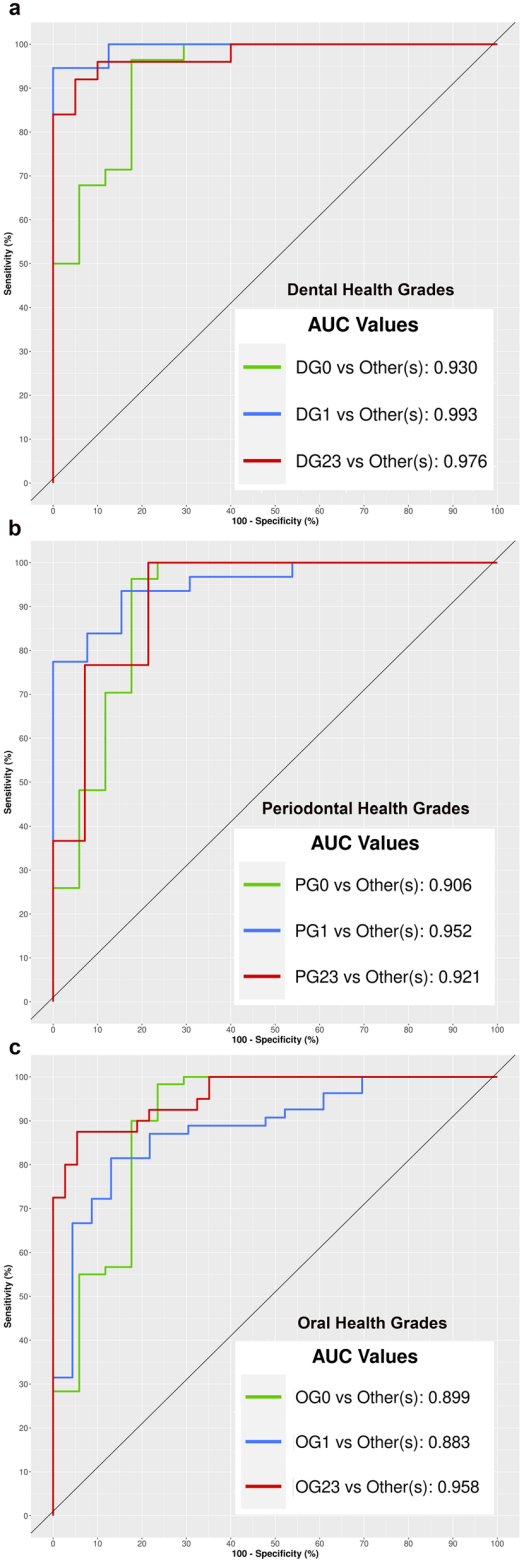
Potential of the salivary microbiome to discriminate the different grades of dental and periodontal health and the combination of both: ROC curves and AUC values (graphics made using the MixOmics package, version 1.6.3).

With regard to the diagnostic value of salivary microbiome, the model obtained to distinguish the grades of the dental subscale was composed of 60 OTUs and the derived AUC values ranged from 0.93 (DG0 *vs* others) to 0.99 (DG1 *vs* others). The model obtained to distinguish the grades of the periodontal subscale was composed of 140 OTUs and the derived AUC values ranged from 0.90 (PG0 *vs* others) to 0.95 (PG1 *vs* others). For the scale of overall oral health, the predictive model was formed by 30 OTUs and the AUC values ranged from 0.88 (OG1 *vs* others) to 0.95 (OG23 *vs* others) (Fig. 7). Focusing on the group with the highest grade of oral pathology (OG23), the predictive potential of PG0_DG23 subgroup and DG0_PG23 subgroup with respect to the others was similar, with AUC values of 0.96 and 0.97, respectively. The contribution of each OTU (value of the coefficient of the variable) that was included in the predictive models of the different clinical conditions is detailed in the supplementary data S5.

## Discussion

The scale of overall oral health employed in this study was first presented by our research team in 2013, with our goal being to develop a scale that produces a single numerical value^18^ to reflect a patient's oral health status, including the presence of dental and periodontal conditions. In this study, the final dental or periodontal subscale grade corresponds to the grades assigned to at least two of the three variables analysed for each subscale. The subscale with the highest grade determined the grade for the scale of overall oral health^18^. To the best of our knowledge, this is the first 16S-based microbiome research to determine the individualised impact of different grades of dental and periodontal disease and the combination of both (hereinafter referred to as oral disease or oral pathology) on the salivary microbiota, evaluating which of them shows the highest impact on this bacterial community.

It should be noted before our main findings are discussed that their difference to previous studies should be interpreted with caution. In particular, it is well-known that each step of the 16S rRNA gene NGS workflow may lead to pitfalls and biases that affect, for example, a study’s design and sample collection, the nucleic acid extraction and sequencing, and the bioinformatic and statistical analyses^64,65^. This lack of a standardised workflow has led to uncertainty regarding the transparency, reproducibility and comparability of 16S-based microbiome research^66^. In the present study, other factors that should also be taken into account are the type of saliva sample analysed (stimulated *vs* non-stimulated)^67^, as well as the different clinical criteria used to define the dental and periodontal health status.

### Impact of the dental and periodontal subscales and the scale of overall oral health on the salivary microbiota: alpha diversity indicators and structure of the bacterial community

Various 16S-based microbiome studies on the impact of dental decay on the salivary microbiota of adults have used the DMFT index to place individuals in a healthy or a caries group^25,68,69^, with the latter including subjects with active caries (DMFT ≥ 6)^68^ or inactive tooth decay^69^. In their study, Rupf et al.^70^ used the Nyvad criteria to define caries activity and recruit patients for their sample who had at least three caries lesions that reached the dentine; they also used the DMFT Index and the ICDAS^70^.

As findings derived from these studies, the saliva samples from the healthy and caries groups generally had similar levels of richness and diversity^25,68–70^. This was true whether the diseased group was composed of subjects with active^68^, inactive^69^ or cavitated^70^ caries. In the present series, the salivary microbiota of those with the highest dental pathology grades (DG23) was significantly richer in bacteria than that of those who had a healthy dental grade (DG0); DG23 also showed a trend towards increased diversity and evenness. It should be noted that the dental health subscale incorporates variables that not only include the number of caries and their severity, but also the levels of supragingival plaque, all of which could affect the bacterial richness of the salivary community.

In most of the papers that characterise the salivary microbiome of adults with various periodontal health statuses, the classification system of the American Academy of Periodontology^71^ is used to obtain diagnoses^26,72–75^. Indeed, only Chen et al.^76^ have used their own diagnostic criteria, which took into account a patient’s probing pocket depth (PPD), clinical attachment loss (CAL), bleeding on probing (BOP) and gingival redness/oedema. In our study series, the periodontal health subscale was defined by levels of gingival inflammation and the number of periodontal pockets and their severity.

There is some inconsistency between the alpha-diversity results of various studies in the literature and our findings, with several of the former describing greater richness^26,76^, diversity^76^ and evenness^26^ in the saliva samples of patients with chronic periodontitis than in those who were periodontally healthy. In our study, however, worsening periodontal health revealed a trend of increased alpha diversity, but these results were not statistically significant for any of the related parameters used in our between-grade comparisons, like ocurred in other series^73^.

Our results for the scale of overall oral health revealed an association between a higher grade and significant increases in the richness and diversity of the salivary microbiota. This was particularly the case for the higher grades, which are determined using a combination of dental and periodontal parameters. In line with the findings of Takeshita et al.^28^, our results demonstrated the potential impact of the simultaneous presence of dental and periodontal disease on the richness and diversity of the salivary microbiota.

It has been found in various studies that the structure of the global salivary microbiota is similar in patients with good oral health^77–79^. This is supported by the findings of our research, where the structure of the salivary bacterial community in the participants with oral health (grade 0) was different to that of the patients with dental, periodontal or oral disease, especially high grades.

### Impact of the dental and periodontal subscales and the scale of overall oral health on the salivary microbiota: composition of the core microbiome and testing differential abundance

Numerous 16S-based microbiome studies on salivary microbiota in the literature have only conducted an analysis of the differential abundance of the taxa associated with various oral conditions^26,29,70,80^. In this study, it is our view that it is essential to analyse salivary microbiota from a dual perspective: the prevalence of the taxa determining the core microbiome; and their differential abundance in relation to the different DGs, PGs and OGs.

The issue of taxonomic assignment is dealt with in some studies of salivary microbiota by using genus as the lowest phylogenetic level^25,26,80^. The 16S rDNA sequencing-based technique, especially from Illumina platforms, lacks the necessary resolution to produce an identification of all OTUS at the species level^81,82^; however, identification at, at least, this level is desirable in the oral microbiota. In our study, we have been able to verify how different species from the same genus are associated with different oral conditions, for example: *Porphyromonas catoniae* was a core species linked to dental and periodontal health, while *Porphyromonas endodontalis* was associated with dental and periodontal pathology. In relation to the differential abundance data, while *Fusobacterium periodonticum* was present in significantly higher numbers in the dentally healthy (DG0), this was only the case for those with high grades of dental pathology (DG23) in *Fusobacterium nucleatum* subsp. *vicentii*. Even, some authors have demonstrated that different strains of the same species of *Veillonella parvula* had different implications for oral health or disease^68,70^. This would justify the claim of Callahan et al.^83^ concerning the biological importance of conducting 16S rRNA gene-based analyses at the bacterial variant level.

In terms of prevalence, core species in the literature on the salivary microbiome have various definitions and, as a consequence, any associated findings are difficult to compare^28,68,72,73^. In this study, the prevalence of grade-specific core species was less than 2.5% (≤ 10 OTUs), that is, they were only present in those with dental or periodontal health, or those with dental or periodontal pathology. While the abundance of the G0 specific core was 8% for dental health and 1% for periodontal health, this figure was around 2.5% for the G23 specific core in both subscales. Interestingly, about 9% (35 OTUs) of the taxa were non-specific core species; in other words, they were present in all the participants, regardless of their dental or periodontal-health grades. Moreover, most of them (about 70%) were common to both subscales, while the non-specific core’s abundance was approximately 50–57%. We demonstrated for the first time that the non-specific core of the salivary microbiome comprises a greater number of species in higher abundances than the specific-core associated with a particular dental or periodontal condition (healthy or pathology). Interestingly, more than half of the non-specific core species in the present series were the same as those previously identified by Takeshita in ≥ 75% of Japanese adults^28^; some of the most predominant species with an abundance ≥ 1% were: *Granulicatella adiacens*, *Haemophilus parainfluenzae*, *Prevotella* sp. (*P. melaninogenica*), *Rothia mucilaginosa*, *Streptococcus* sp. (*S. salivarius*), and *Veillonella* sp. (*V. parvula*). These results confirm that several bacterial taxa in the salivary microbiome could be present in ethnically diverse populations, regardless of their dental and periodontal health statuses.

In terms of our differential abundance findings, the number of taxa present in the salivary microbiota at significantly different levels for the subscale or overall scale grades did not exceed 25%, and mainly included non-core species (5–16%); for the oral health scale, the figure was 22%. This number of taxa was higher for the dental than the periodontal subscale (25% *vs* 7% of the total OTUs). This is the first time that this finding has been reported in the literature. If it is assumed that the salivary microbiome comprises a mix of bacterial communities originating from various sites in the oral cavity^67^, our observations provided evidence that the relative abundances of the most predominant bacteria in saliva are not strongly related to the grade of overall oral health. This corroborates the view that the dominant source of the salivary microbiome is probably the bacterial communities on the mucosal surfaces, especially those from the dorsum of the tongue^67,84,85^.

When we compared our two subscales, we detected that parameters like supragingival plaque levels and the number and severity of caries had a greater differential abundance impact on the salivary microbiota than periodontal parameters such as levels of gingival inflammation and the number and severity of the periodontal pockets. Consequently, it is clear that supragingival dental conditions affect the abundance of salivary microbiota more than subgingival periodontal conditions, with the former having a greater influence on oral health.

Nevertheless, in the present series, specific bacteria were involved in different oral conditions. In our view, for a species to be evaluated as a possible bacterial biomarker associated with a given oral condition, it must be: predominant in most individuals (core species), especially in those with the oral condition with which there is an intended association; and present in high levels of abundance. Applying these criteria to our 16S-based data, the main bacteria associated with dental health (2–3-fold increment in relative abundance in DG0) were: *Porphyromonas pasteri*, *Fusobacterium periodonticum* and *Veillonella parvula*; for periodontal health (1.69-fold increase in the relative abundance in PG0), the species was *Haemophilus parainfluenzae*. All of these bacteria continued to be associated with overall oral health (1.5–2-fold increase in relative abundance in OG0). The exception was *Fusobacterium periodonticum*, probably because it has been found to be more abundant in patients with periodontitis^27^. These outcomes are in accordance with the findings of most 16S-based studies concerning the identification of the above-mentioned taxa as core species present in > 70% of people^28,68,72,79^. Other authors have, like us, also detected differential abundances of these species, which provide support for dental or periodontal health^27,68–70,76^.

In our study, the main bacteria associated with high grades of dental pathology (2.5–3.5-fold increment in relative abundance in DG23) were: *Streptococcus parasanguinis* clade *411*, *Alloprevotella* sp. *HMT 308*, *Atopobium* sp., and *Fusobacterium nucleatum*_subsp._*vincentii*. Although most of these bacteria have been described as core species in other studies^28^, there is no link to dental pathology in relation to differential abundance. In the periodontal subscale, *Tannerella forsythia* was the only species associated with high periodontal pathology grades (6-fold increment in relative abundance in PG23). This bacterium has also been identified in the core microbiome in our previous studies^27,74^. However, although it was abundant in the subgingival samples taken from participants with periodontitis^75^, these levels in the salivary microbiota were not high (< 0.3%) compared to those of the species mentioned previously. With respect to the other two species that compose the red complex, *Porphyromonas gingivalis* and *Treponema denticola*, although they did not present differential abundance, these taxa were ten and six times more abundant, respectively, in the highest grades of periodontal pathology than in periodontal health.

Only *Fusobacterium nucleatum*_subsp._*vincentii* continued to be the taxon associated with the highest levels of oral pathology, with a 3-fold increase in OG23. Interestingly, although not a core species, *Porphyromonas gingivalis* was nine times more abundant for the highest oral pathology grades, revealing its role not only in periodontal but also in dental pathology, as reported in the literature^86,87^.

### Impact of the dental and periodontal subscales and the scale of overall oral health on the salivary microbiome: co-occurrence network patterns and discriminatory potential of the salivary microbiome

The analysis of networks representing microbe-microbe interactions has increased our knowledge of how microbes potentially interact in their environment^88^. Despite the importance of this type of investigation, our review found that co-occurrence results were reported in only a few papers that evaluated the salivary microbiota of patients with and without caries, the periodontally healthy and those with periodontitis, and adults with different oral health conditions^25,69^.

It is important to note that any comparison of the co-occurrence results in the different studies in the literature should be undertaken with caution: the findings obtained may well be affected by methodological differences such as the different correlation values employed as cut-off points^89^ or the use of different keystone OTU definitions^61^.

The positive and negative correlations detected in a co-occurrence network can describe the tendency of different species to co-occur in a variety of circumstances. Consequently, two species exhibiting a significantly positive correlation in relation to abundance could be a direct indication of a shared preference for a particular combination of environmental conditions; alternatively, it might be a true ecological interaction where, for example, two species can grow better due to metabolite exchanges. In contrast, a negative correlation between species may indicate a competitive interaction for nutrients or differences in physiological requirements to the extent that they never occupy the same niche^90^. As co-occurrence networks are undirected and weighted, there is no directionality of the interactions to the degree that the underlying cause of such patterns cannot be explained^90^.

In our study, like the findings of previous authors^25,69^, the topological characteristics of the salivary microbiome networks differed between the statuses of oral health and the presence of high grades of oral or dental disease. The health-associated network presented as a bacterial community with more interconnections between its members and a greater balance between the coactive and competitive interactions. The disease-associated network was less dense and synergistic exchanges predominated, suggesting that the antagonism between the oral bacteria was not a major driving force in the formation of the disease-associated microbial community^26^. However, in contrast, high grades of periodontal disease did not affect the characteristics of the salivary microbiome co-occurrence network.

One of the most useful features of a co-occurrence network analysis is that hubs or keystone OTUs, which are highly associated taxa in a microbiome, can be identified^91^. However, in the salivary microbiome studies in the literature that included co-occurrence network analyses, attempts to detect keystone OTUs were uncommon^26^.

In the present study, seven of the 12 keystone OTUs identified (58.3%) were part of the salivary core microbiome. In this sense, it has been suggested that the contribution of keystone taxa will be greater if they are part of the core microbiome and consistently present, highlighting the importance of such taxa for microbiome functioning^92^. On the other hand, eight and 10 of the 12 keystone OTUs identified (66.6% and 83.3%, respectively) were low-abundance species (< 1%) with no associated differential abundance results. This confirms that keystone OTUs could have an impact on microbiome functioning, irrespective of the abundance parameters^61^.

Our results revealed that the keystone OTUS identified in the main modules of the networks varied between the clinical groups. This suggests that the possible drivers of the salivary microbiome’s structure and functioning differ in relation to oral health and disease, and even vary in different types of disease. The exception was the species *Porphyromonas endodontalis*, which was identified as a keystone OTU in the OGO and PG23 networks, making it particularly important. Given our findings, and despite the well-known limitations, we believe it is vital to conduct identifications at the species level as part of a co-occurrence network analysis. This is because we identified species in the present networks that were associated with different clinical conditions but belong to the same genus: *Porphyromonas* in OG0 and PG23, *Prevotella* in OG0, OG23 and DG23, and *Fusobacterium* in OG23 and DG23. However, evidence derived from further experimentation is required before hub taxa in inferred networks can be classified as keystone^93,94^.

Our review of the relevant literature identified only a few studies that attempted to evaluate the diagnostic accuracy of the salivary microbiome for diagnosing oral diseases like periodontitis^72,76^. Moreover, to the best of our knowledge, our research is the first to assess the potential of the salivary microbiome for distinguishing different grades of dental and periodontal disease, or a combination thereof. The results we obtained revealed the excellent discriminatory power (AUC values > 0.88) of this approach. In line with the differential abundance results, a higher number of predictive variables (140 OTUs) was required to discriminate the periodontal condition than the dental condition (60 OTUs). Interestingly, the best model of the salivary microbiome, i.e., the one associated with the lowest number of predictor variables (30 OTUs), was the model for a combination of dental and periodontal disease, evidencing the impact of both of these conditions on saliva. Focusing on the group with the highest grade of oral pathology (OG23), the heterogeneity of this group (based mainly on the presence of two subgroups, DG0_PG23 and PG0_DG23) did not affect the discriminatory power of the salivary microbiome.

The main limitation of this study is clinical, due to the difficulty in recruiting patients with the highest level of periodontal pathology. This meant that grade 2 and grade 3 patients had to be placed in the same group, despite our preference to conduct an analysis for each grade individually. On the other hand, we are aware that relative abundance measurements derived from 16S rRNA gene amplicon sequencing do not accurately reflect absolute taxon concentrations. This would require, at least, one broad-range qPCR determination for the calculation of inferred bacterial concentrations, which are a reasonable proxy of species-specific qPCR values^95^. Although this was not an objective in the present study, another interesting avenue for future research would be to evaluate the impact that smoking has on the salivary microbiota of patients with different grades of dental, periodontal or oral health.

In conclusion, the simultaneous presence of dental and periodontal pathology has a potentiating impact on the richness and diversity of the salivary microbiota. The structure of the bacterial community in oral health differs from that present in dental, periodontal or oral disease, especially in high grades. The non-specific microbiome core comprises a greater number of species present in higher abundance than the specific core of a particular dental or periodontal condition (health or pathology). The number of taxa in the salivary microbiota with differential abundances between the DGs, PGs or OGs represents, at most, a quarter of the bacterial community and are mainly non-core species. Supragingival dental parameters condition the abundance of the microbiota more than subgingival periodontal parameters, with the former contributing more to the impact that oral health has on the salivary microbiome.

The oral health-associated network has a bacterial community with more interconnections between its members, and a greater balance between its synergistic and competitive interactions, than the network associated with high grades of oral or dental disease; meanwhile, high grades of periodontal disease do not condition the characteristics of the salivary microbiome co-occurrence network. The possible keystone OTUs are different in relation to oral health and disease, and even these vary in different types of disease: half of them belongs to the core microbiome and are independent of the abundance parameters.

The salivary microbiome, involving a considerable number of OTUs, shows an excellent discriminatory capability for distinguishing different grades of dental, periodontal or oral disease; considering the number of predictive OTUs, the worst model is that which predicts the periodontal status and the best model is that which predicts the combined dental and periodontal status.

## Supplementary Information

Supplementary Table S1.

Supplementary Table S2.

Supplementary Table S3.

Supplementary Table S4.

Supplementary Table S5.
